# Quantitative evaluation of ankle cartilage in asymptomatic adolescent football players after season by T2-mapping magnetic resonance imaging

**DOI:** 10.1186/s12938-021-00970-9

**Published:** 2021-12-28

**Authors:** Sipin Luo, Yi Cao, Peng Hu, Nan Wang, Yeda Wan

**Affiliations:** Department of Radiology, Tianjin Hospital, Tianjin University, #406 Jiefangnan Rd., HeXi district, Tianjin, 300299 People’s Republic of China

**Keywords:** Ankle, Cartilage, Magnetic resonance imaging, Football player, T2-mapping

## Abstract

**Background:**

Ankle sprain affects the structure and function of ankle cartilage. However, it is not clear whether the daily training and competition affect the ankle cartilage without acute injury. Changes in ankle cartilage without injury may influence future strategies to protect ankle function in athletes. This study aimed to evaluate whether the composition of ankle cartilage significantly altered in asymptomatic adolescent football players after a whole season of training and competition using T2-mapping magnetic resonance imaging (MRI).

**Materials and methods:**

12 local club’s U17 asymptomatic adolescent football players without abnormalities in routine MRI were included. Routine and T2-mapping MRI were performed to measure the cartilage thickness of tibiotalar joint (TT) and posterior subtalar joint (pST) and T2 values in pre- and post-seasons. All of them took the right side as dominant foot.

**Results:**

In the pre- and post-seasons, cartilage T2 values in TT (talus side) and pST (calcaneus side) were higher than that of TT (tibial side) and pST (talus side) (all *p* < 0.05), which was caused by magic angle effect and gravity load. No statistically significant differences in thickness after season in the other cartilages of ankle were found compared with that before the season (all *p* > 0.05). However, T2 values of TT (tibial side and talus side) cartilage in the dominant foot were significantly reduced after season (*p* = 0.008; *p* = 0.034). These results indicate that the microstructure of articular cartilage changes in the joints with greater mobility, although no trauma occurred and the gross morphology of cartilage did not change.

**Conclusion:**

Changes in the T2 values of tibiotalar joint cartilage in the dominant foot of healthy young athletes before and after the season suggest that the microstructure of cartilage had changed during sports even without injury. This finding suggests that the dominant ankle joint should be protected during football to delay degeneration of the articular cartilage.

## Introduction

Football is one of the most popular sports in the world, with approximately 265 million players worldwide, including professional and amateur players [1]. The ankle joint is the most common injured area in athletes, and ankle sprain accounts for almost 45% of all sports injuries [2] and 80% of football injuries [3]. A long-term study of more than 3000 professional players in the Union of European Football Association (UEFA) elite clubs showed that the risk of isolated syndesmotic injuries of the ankle joint is increasing [4]. In the training of adolescent football players, about half of the players who suffered from ankle injuries are absent from training for less than 1 week, one-third for 1–4 weeks, and 10 to 15% for longer [5]. The absence of competition and training affects the training level of young athletes. On the other hand, 70% of ankle osteoarthritis occurs after ankle injury [6]. Football players are 5 to 12 times more likely to be prone to osteoarthritis in the ankle than the general population and it occurs 4 to 5 years earlier [7]. The occurrence of arthritis is related to the decrease of ankle stability caused by direct ankle injury or the damage of surrounding supporting structure [7]. Early-onset of arthritis will shorten the football players’ career and has adverse effects on the health of retired players [8].

Imaging examination can clarify the bone and ligament condition of ankle joint injury [9]. The condition of ankle joint cartilage, tendon, and ligament can be observed by magnetic resonance imaging (MRI) at the same time. According to the different clinical conditions, scanning sequences and directions can be optimized and a contrast agent is introduced to find hidden lesions [10]. Ankle injuries can be graded on MRI, and high-grade ankle sprain with cartilage defect can prolong the time to return to competition [11, 12]. In addition, the ankle joint tissues can be quantitatively evaluated by MRI, such as T2-mapping sequence which can show water and collagen content and biochemical integrity in cartilage [13]. T2-mapping sequence can also distinguish the layers of articular cartilage and measure T2 value accurately [14]. T2-mapping sequence is easy to apply in any direction and is considered to be the most useful component analysis MRI sequence in sports medicine [15].

Taken together, the present study suggests that ankle sprains affect the structure and function of ankle cartilage in football players. However, in the absence of acute injury, whether the ankle cartilage changes after training and competition has not been determined. If the ankle cartilage is altered in the absence of injury, it will affect the development of strategies to protect the athlete’s ankle function. Therefore, this study is the first to use T2-mapping MRI to continuously examine the ankle joint of youth football players, and evaluate whether the microstructure of ankle cartilage changes after the whole season of training and competition.

## Results

### Study population

Twelve players who were recruited before October 2017 and kept training for more than 1 year included 2 forwards, 4 midfielders, 5 defenders, and 1 goalkeeper, and all of them took the right side as dominant foot, which met the study criteria. All players have the same strength and physical training methods. The professional training content is the same except for goalkeepers. The amount of training varies according to different positions on the field. They would be 17 years old at the end of 2019 (Table 1). The team finished Chinese Huabei area regular games in early August and failed in entering the knockout round of the whole nation.

**Table 1:** General information of 12 players

No.	Age	Height (cm)	Join team data	Position	Dominant foot
1	17	165	2017.10	FW	Right
2	17	169	2017.10	FW	Right
3	17	172	2017.10	RM	Right
4	17	165	2017.10	RM	Right
5	17	166	2017.10	CM	Right
6	17	168	2017.10	RM	Right
7	17	166	2017.10	CB	Right
8	17	172	2017.10	CB	Right
9	17	171	2017.10	CB	Right
10	17	165	2017.10	RB	Right
11	17	168	2017.10	RB	Right
12	17	178	2017.10	GK	Right

Age calculated until December 31, 2019

### Quantitative analysis

#### Tibiotalar joint (TT) cartilage

**Fig. 1:**
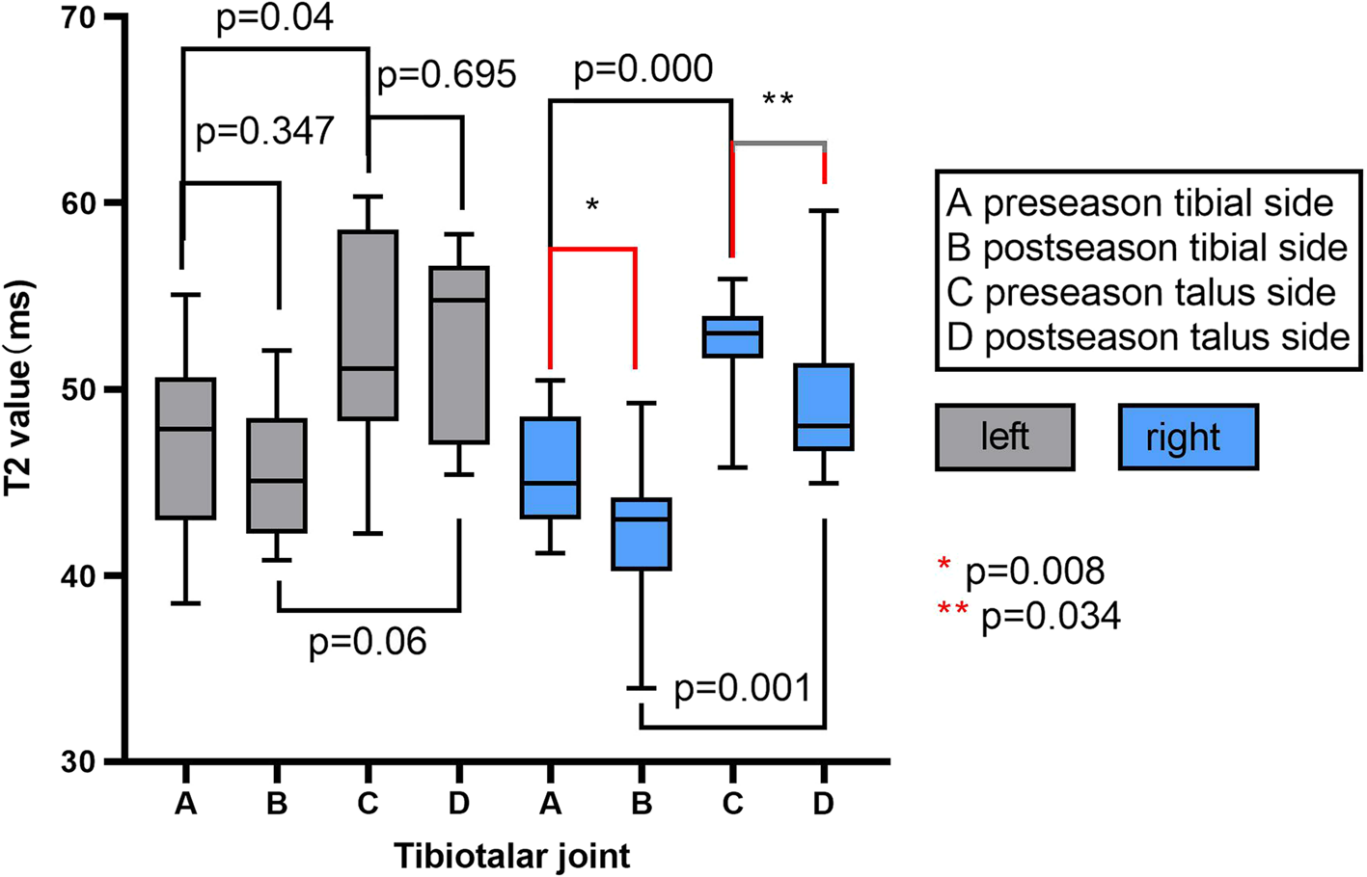
Average cartilage T2 values of tibiotalar joint in pre- and post-season. Right side was dominant

**Fig. 2:**
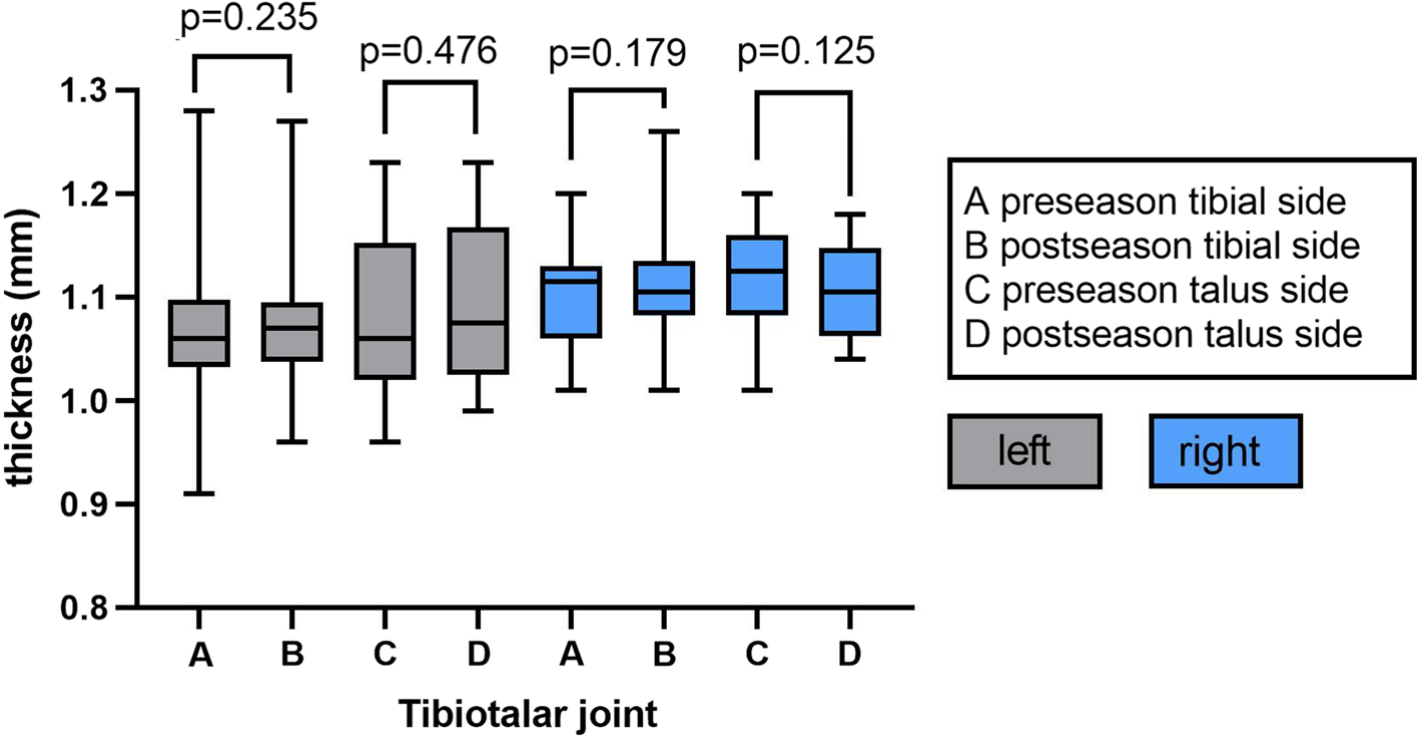
Average cartilage thickness of tibiotalar joint in pre- and post-season. Right side was dominant

In pre-seasons, the average T2 values in TT (tibial side) cartilage of right (the dominant foot) and left ankle was 45.38 ± 3.05 and 46.98 ± 4.99 ms, and the average thickness was 1.10 ± 0.05 mm (right) and 1.06 ± 0.08 mm (left), while the average T2 values of TT (talus side) cartilage was 52.57 ± 2.61 ms (right) and 52.36 ± 5.78 ms (left), and the average thickness was 1.12 ± 0.05 mm (right) and 1.08 ± 0.08 mm (left). In post-seasons, the average T2 values in TT (tibial side) cartilage of right and left ankle was 42.51 ± 3.87 ms and 45.43 ± 3.65 ms, and the average thickness was 1.11 ± 0.06 mm (right) and 1.075 ± 0.07 mm (left), while the average T2 values of TT (talus side) cartilage was 49.33 ± 3.98 ms (right) and 52.18 ± 5.09 ms (left) and the average thickness was 1.10 ± 0.05 mm (right) and 1.09 ± 0.07 mm (left) (Figs. 1 and 2).

The cartilage T2 values in the talus side of TT in both ankles were higher than those of the tibial side in pre-season or post-season (all *p* < 0.05). In pre-season vs post-season comparison, the cartilage T2 values were decreased in both tibial (*p* = 0.008) and talus side (*p* = 0.034) of the dominant foot’s TT joint, while the average thickness and the T2 values of TT cartilage in the left ankle showed no significant change (all *p* > 0.05) (Figs. 1 and 2).

#### Posterior subtalar joint (pST) cartilage

**Fig. 3:**
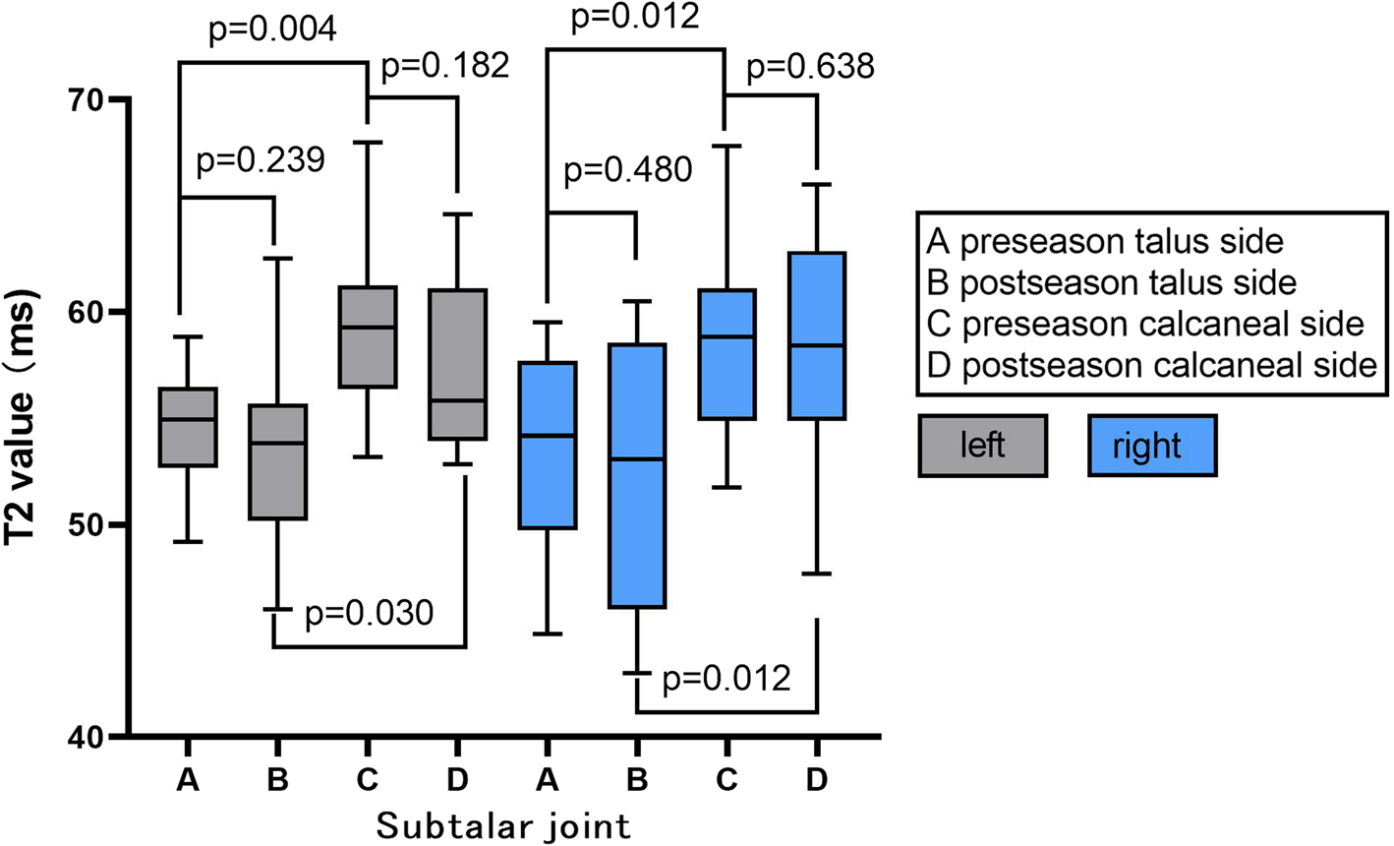
Average cartilage T2 values of subtalar joint in pre- and post-season. Right side was dominant

**Fig. 4:**
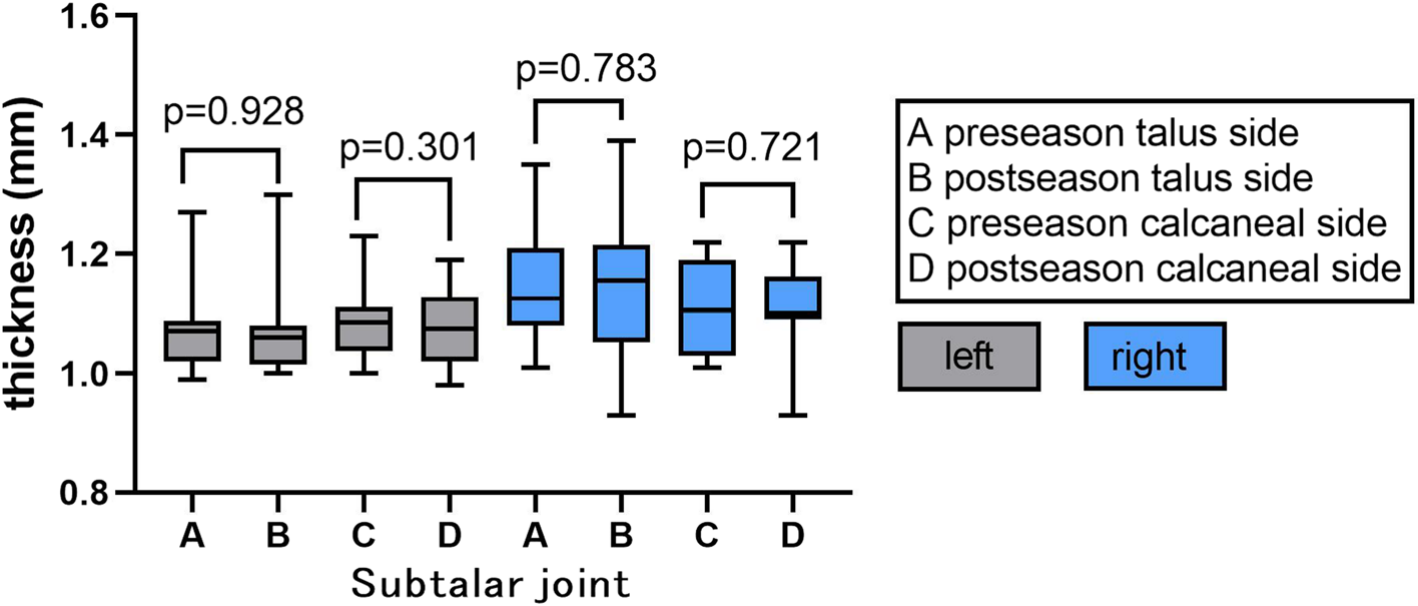
Average cartilage thickness of subtalar joint in pre- and post-season. Right side was dominant

In pre-seasons, the average T2 values in pST (talus side) cartilage of right (the dominant foot) and left ankle was 53.45 ± 4.56 ms and 54.68 ± 2.85 ms, and the average thickness was 1.15 ± 0.09 mm (right) and 1.07 ± 0.07 mm (left), while the average T2 values of pST (calcaneus side) cartilage was 59.02 ± 4.89 ms (right) and 59.60 ± 4.31 ms (left) and the average thickness was 1.11 ± 0.08 mm (right) and 1.08 ± 0.06 mm (left). In post-seasons, the average T2 values in pST (talus side) cartilage of right and left ankle was 52.07 ± 6.2 ms and 53.24 ± 4.51 ms and the average thickness was 1.14 ± 0.11 mm (right) and 1.07 ± 0.08 mm (left), while the average T2 values of pST (calcaneus side) cartilage was 58.5 ± 5.14 ms (right) and 57.38 ± 4.08 ms (left) and the average thickness was 1.10 ± 0.08 mm (right) and 1.07 ± 0.06 mm (left) (Figs. 3 and 4).

The cartilage T2 values in the calcaneus side of pST in both ankles was higher than those of the talus side in pre-season or post-season (all *p* < 0.05). In pre-season vs post-season comparison, the cartilage T2 values in dominant foot’s pST joint and non-dominant foot’s TT and pST joints had no significant changes (all *p* > 0.05), as well as cartilage thickness (all *p* > 0.05) (Figs. 3 and 4).

## Discussion

The purpose of this study was to analyze microstructure changes in ankle cartilage of asymptomatic young football players by comparing T2 values before and after season.

Articular cartilage is composed of chondrocytes and extracellular matrix. Extracellular matrix is mainly composed of proteoglycan, collagen, and water, which account for 90% of articular cartilage. The collagen arrangement (type II predominated) is relevant to the cartilage layer. Water is mostly free water, which plays a role in the conduction load in cartilage. MRI T2 relaxation time of articular cartilage is mainly related to the structure and content of extracellular matrix and the stress of cartilage. T2-mapping sequence is applied to measure MRI signal intensity at different echo time and to calculate T2 values using multi-echo spin-echo sequence [16].

In this study, we found that the cartilage T2 values of the talus side were higher than those of tibial side in TT, and T2 values of calcaneal side were higher than those of talus side in pST in both pre- and post-season whether the foot was dominant or not. Lockard et al*.* found that the average T2 values of ankle cartilage and hind foot were significantly different in normal volunteers [17]. Our results of TT cartilage were consistent with previous study, which might be due to the magic angle effect and the different gravity load [18, 19]. We found the same pattern in pST, which showed different angles to main magnetic field direction from the TT. We considered this phenomenon to be physiological. Gravity load in the lower parts of cartilage was larger than that in the upper parts due to weight-bearing. Since the schedule and intensity of youth league matches are lower than that of adult league, the body conditions of players do not change much before and after the season. We only collected the information of height and weight of players before the season, but did not compare the changes after the season. Because tibiotalar and subtalar joint have different angles relative to the main magnetic field, we believe that the magic angle effect is less important than the gravity load for this result. T2 values in the lower parts of cartilage were higher, but there was no bone edema in asymptomatic adolescent players, indicating increasing water content and physiological changes in microtrauma repair of cartilage. It is necessary to identify whether these pathological changes in the cartilage structure caused by the phenomenon are related to age, overweight, or other factors after long-term accumulation in further study.

In this study, we found for the first time that after a season of training and competition, the thickness of the ankle cartilage did not change significantly, while the T2 values of TT (tibial side) and TT (talus side) cartilage of the dominant foot of healthy young athletes decreased significantly compared with the pre-season. This suggests that the microstructure of TT cartilage of the dominant foot changes after a season of training and competition, even without acute injury. In football, the activity of the customary foot is greater, and the rotation, flexion and extension functions required by various technical movements are easily affected by external forces in all directions. The non-dominant foot acts primarily as support and is driven primarily by the direction of gravity. We believe that multiple external forces cause changes in the physiological structure of the articular cartilage of the dominant foot, which can be quantitatively detected by T2-mapping MRI. The volume of articular cartilage is positively correlated with height and weight in normal people, but decreases with the course of osteoarthritis [20, 21]. In our study population, healthy adolescent football players who met the inclusion criteria had normal ankle function, and the general shape of the ankle joint cartilage had no obvious changes. Previous study showed that T2 values of hyaline and fibrous cartilage decline after running in the knee articular cartilage and meniscus of normal volunteers, indicating that sports affect the normal cartilage molecular structure and molecular biological variety [22]. Cartilage defect and ligament injury were found in routine MRI in the observation of adult professional athletes with subchondral bone marrow edema. Behzadi et al*.* found that T2 values of TT are higher than those in healthy volunteers [23]. It was found that the asymptomatic adolescent football players did not suffer from subchondral bone marrow edema, cartilage defect, and ligament injury signs in routine MRI. The T2 values of TT cartilage in the dominant foot were decreased but did not change in pST and non-dominant foot’s tibiotalar and posterior subtalar joint after season. In magnetic resonance imaging, the T2 value of a substance is determined by the position of hydrogen atoms and the uniformity of the surrounding magnetic field. When two hydrogen atoms are close to each other, the uniformity of the surrounding magnetic field decreases, resulting in a shortened relaxation time and a shortened T2 value. The disordered arrangement of collagen fibers in cartilage leads to an increase in local magnetic field inhomogeneity and ultimately to a shortening of T2 values. Compared with the adult professional players, the competition intensity of adolescent games was more weakness and the probability and degree of acute ankle injury were lower [1], so we believe that articular cartilage structure changes with exercise even in the absence of injury. On the other hand, the activity of tibiotalar joint, mainly participating in ankle rotation, flexion, and extension, was greater than that of posterior subtalar joint, suggesting that this joint was more susceptible to sports. Compared with the dominant foot, the opposite one mainly played a supporting role in completing technical actions, which was less active. We considered that these factors might explain the results.

The condition of bone, cartilage, ligament, and tendons can be estimated by various medical imaging methods and graded by MRI performances when injury occurs [9, 11]. Previous study showed that high-grade ankle sprain with cartilage defect is a risk factor that leads to a delay in return to competition [12]. In our study, it was shown that decreased cartilage T2 values of TT in the dominant foot were observed in normal adolescent football players at the end of season. Based on the above results, it can be concluded that during the career of football players, the articular cartilage of ankle used by football players will first undergo subtle structural changes. Acute trauma or long-term chronic wear leads to inflammatory cell infiltration, aggravating degeneration of articular cartilage, resulting in thinning or even disappearance of cartilage thickness, and osteoarthritis in advance, which reduces athletes’ athletic ability and makes them unable to adapt to fierce competition and retire. Therefore, during football training, the dominant ankle joint of healthy players should be consciously tested and evaluated regularly, and more detailed protective methods should be developed to delay cartilage microstructure changes, rather than waiting for ankle discomfort or trauma to intervene.

There were still some limitations in this study. First of all, the sample size was small. Among the football clubs participating in the top league in local cities, only one U17 team can guarantee professional football training and regular matches, so there may be sampling error in our results. Since the league sets a home and away double-cycle match, it is difficult to track the visiting team. In the future, multi-center studies can be carried out to verify our results. Secondly, our study only focused on the data at the beginning and end of a season, and did not determine whether T2 values of cartilage recovered through off-season recovery before the start of the next season. Thirdly, we only measured the average T2 values in whole TT and pST cartilage, the differences between the shallow layer and the deep layer were ignored. In future studies, better gradient coils can be used to obtain higher resolution images, and more accurate results can be obtained by comparing the cartilage of different layers.

## Conclusion

The higher T2 values in the lower parts of tibiotalar and posterior subtalar joint cartilage might be due to the magic angle effect or inconsistent cartilage weight load. Training and competition had an influence on the dominant foot, leading to a significant decrease in TT cartilage T2 values and prompting that the dominant foot was greatly influenced by competitive activities and should be cared for in daily training to avoid cartilage damage. T2-mapping MRI had the ability to detect early cartilage changes in the ankle of adolescent football players.

## Materials and methods

### Study population and inclusion/exclusion criteria

The members of U17 adolescent team (season 2019, from March to September) of local professional football club who participated in the 2018–2019 season of Chinese Super League (CSL) were included in the study. Before the season, quantitative MRI including T2-mapping and routine T1-weighted imaging (T1WI) and fat-suppressed proton density-weighted imaging (fsPdWI) sequence was performed for the players who met the inclusion criteria. Repeat MRI scans of the suitable players were taken within a week after the end of the season. Before MRI, they needed to rest for more than 24 h. The players who had an acute slight ankle injury [24] during off-season training or match resulting in continuous absence for more than 7 days, ankle pain or other symptoms in ankle joint at present, recurrent minor ankle injury, serious ankle injuries at the past/during the match or other reasons resulting a long-term absence were excluded. Apart from the above principles, players who were found to have ankle cartilage defects, subchondral bone marrow edema, abnormal ligament, and tendon shapes or signals by routine T1WI and fsPdWI were excluded. All participants volunteered and agreed to participate in the study since they were informed that the examination was noninvasive and free of harmful factors such as radiation as well as their professional training, technical statistics and team secrets were not collected. This study was approved by the ethics committee at Tianjin Hospital, Tianjin University (approval number: 2020-162).

### Patient and public involvement

Patients or the public were not involved in the design, conduct, reporting, or dissemination plans of this research.

### Image acquisition

A 3.0-T MR system (MR750, GE Healthcare, Milwaukee, WI, USA) was applied for data acquisition including routine T1WI, fsPdWI, and T2-mapping sequence. All the participants were placed in the supine position with their feet entering first and ankle coil (8-channel). Detailed parameters are listed in Table 2.

**Table 2:** Parameters of MRI sequence

	T1WI	fsPdWI	T2-mapping
Orientation	Sag/o-Tra	Sag/Cor/o-Tra	Sag
TR (ms)	694	2700	1000
TE (ms)	13.3	25	9/18/27/36/45/54/63/72
FOV (cm)	20	18	16
Frequency	320	320	256
Phase	192	160	128
Bandwidth	31.25	50	50
ETL	4	16	/
Nex	2	2	2
Slice thickness (mm)	4	Sag/o-Tra:4, Cor:3.8	4
Gap (mm)	1	1	1
slices	13	Sag/o-Tra:13, Cor:14	13

o-Tra: 20° to the horizontal

### Image analysis

**Fig. 5:**
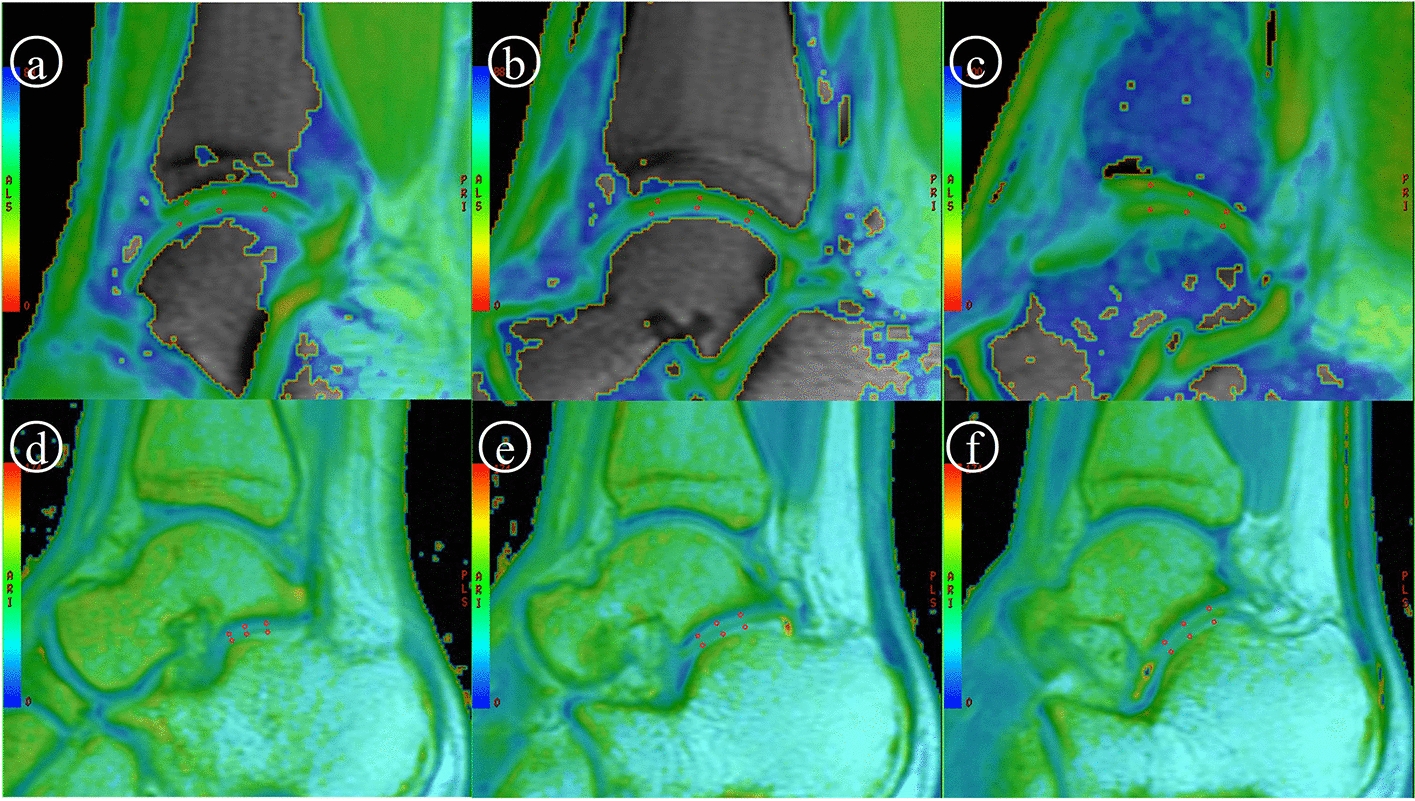
The T2 values calculated by GEAW workstation are used to create pseudo-color images, respectively. 1 mm^2^ ROI was set on cartilage to lateral to measure T2 values. Cartilage thickness was measured at same position in Sag-T1WI (not list). Due to different thresholds, different colors represent different T2 values. **a**–**c** The T2 values of TT cartilage. ROI was placed on articular cartilage of calcaneus side and talus side, respectively. Blue–red shows high–low T2 values. **d**–**f** The T2 values of pST cartilage. ROI was placed on the articular cartilage of the side of the talus and the side of the calcaneus, respectively. Red–blue shows high–low T2 values

Two radiologists with different seniority (5 years and 21 years) read the MRI scan in PACS in a double-blinded manner. The players who had abnormal MRI manifestations in routine T1WI and fsPdWI were eliminated according to the exclusion criteria. Disagreements were discussed after independent diagnosis. Subsequently, referred to ankle anatomic structures in T1WI, the locations of the ROIs (region of interest) focused on the articular cartilage of tibiotalar joint (TT) and posterior subtalar joint (pST) were discussed and decided by those two radiologists together. The measurements were performed by a radiologist (5 years seniority). Reconstruction and measurement of T2-mapping were processed by GE AW 4.6 workstation with function tool 9.4.05 software (Copyright belongs to GE Healthcare, Milwaukee WI, USA). Six circular ROIs with 1 mm^2^ area were located on TT and pST cartilage from anterior to posterior and lateral to medial, respectively, referred to as sagittal T1WI. The cartilage thickness in these locations in T1WI was also measured. Figure 5 shows the ROI locations.

### Statistical analysis

The average T2 values and thickness of s and pST (talus and calcaneus side) cartilage were calculated according to those 6 areas in cartilage, respectively. First, we compared the pre-season T2 values of TT (tibial side vs talus side) and pST (talus side vs calcaneus side) cartilage with Mann–Whitney U test, and the same statistical method was used for post-season data. After that, we compared the T2 values and thickness between seasons (pre-season vs post-season) with Wilcoxon signed-rank test. The above data were divided into the dominant side and non-dominant side, for comparison. All data were analyzed by SPSS 22.0; *p* < 0.05 was regarded as statistical significance.

## Data Availability

Not applicable.

## Abbreviations

TT: Tibiotalar joint
pST: Posterior subtalar joint
MRI: Magnetic resonance imaging
ms: Millisecond
UEFA: Union of European Football Association
CSL: Chinese Super League
T1WI: T1-weighted imaging
fsPdWI: Fat-suppressed proton density-weighted imaging
